# A New Perspective: Mitochondrial Stat3 as a Regulator for Lymphocyte Function

**DOI:** 10.3390/ijms19061656

**Published:** 2018-06-04

**Authors:** Mercedes Rincon, Felipe Valença Pereira

**Affiliations:** Department of Medicine, Immunobiology Division, University of Vermont, Burlington, VT 05405, USA; fvalenap@uvm.edu

**Keywords:** Stat3, mitochondria, mitochondrial Stat3, calcium, ATP, CD4 cells, B cells, CD8 cells, T cells

## Abstract

Stat3 as a transcription factor regulating gene expression in lymphocytes during the immune response is well known. However, since the pioneering studies discovering the presence of Stat3 in mitochondria and its role in regulating mitochondrial metabolism, only a few studies have investigated this non-conventional function of Stat3 in lymphocytes. From this perspective, we review what is known about Stat3 as a transcription factor and what is known and unknown about mitochondrial Stat3 (mitoStat3) in lymphocytes. We also provide a framework to consider how some of the functions previously assigned to Stat3 as regulator of gene transcription could be mediated by mitoStat3 in lymphocytes. The goal of this review is to stimulate interest for future studies investigating mitoStat3 in the immune response that could lead to the generation of alternative pharmacological inhibitors of mitoStat3 for the treatment of chronic inflammatory diseases.

## 1. Introduction: Revisiting the Role of Stat3 25 Years Later

**Figure 1:**
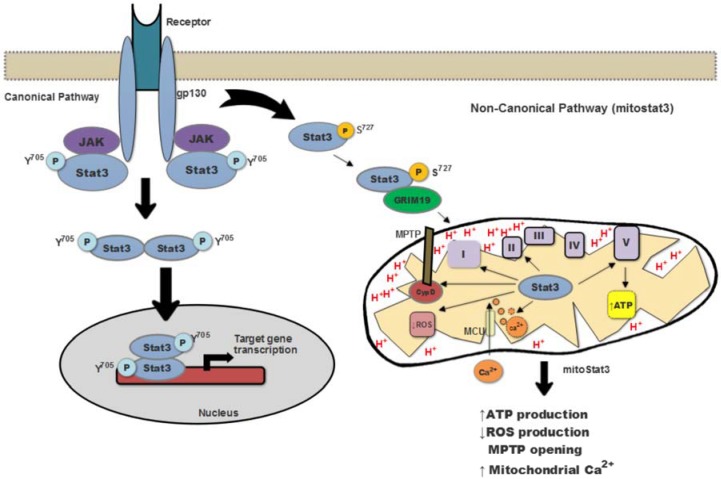
Canonical and non-canonical Stat3 signaling pathways. In the canonical pathway, Stat3 is translocated to the nucleus and induce specific gene expression. Non-canonical signaling Stat3 is imported into mitochondria via GRIM-19. mitoStat3 regulates ATP synthesis, decreases ROS release, increases mitochondrial Ca^2+^ and MPTP opening.

Stat3 was initially identified in the early 1990s, when most of the purification and cloning of transcription factors first took place [1,2]. Stat3 was originally found to be a transcription factor bound to genes regulated by IL-6 in hepatocytes. Stat3 is expressed almost ubiquitously in most cells, but a number of studies showed that it is “activated” by the phosphorylation of Tyr^705^ by specific members of the Jak/Tyk2 family of tyrosine kinases (Jak1, Jak2, and Tyk2). Tyr phosphorylation causes dimerization and translocation from the cytosol to the nucleus where Stat3 binds to specific gene promoters and regulates gene expression [3,4] (Figure 1). This initial, simplistic model of regulation of Stat3 has since become more complex with the contribution of numerous studies, as described in other reviews in this issue. In addition to its regulation by phosphorylation on Tyr^705^, Stat3 is also regulated by phosphorylation on Ser^727^ by some members of the MAP kinases. Stat3 is regulated by cytokines others than IL-6 that share the gp130 signal transducer in their receptors (e.g., onconstatin M, IL-11, IL-27) [2,5]. Other stimuli such as IL-10, IL-21 and leptin do not share gp130 to activate Jak/Tyk kinases, but they can also activate Stat3 [6,7,8]. However, the best activator of Stat3 remains IL-6 in part due to the higher affinity of gp130 to IL-6R [9]. Stat3 activity as transcription factor is also regulated by phosphatases as well as by the Suppressors of Cytokine Signaling (SOCS), which can interfere with nuclear recruitment or promote Stat3 degradation [10]. Considering the regulation of Stat3 activity by IL-6 and other cytokines and the relevance of these cytokines in the immune response, a number of functions in T and B lymphocytes have been reported to be regulated by Stat3 as a transcription factor (further discussed below).

Although it has been more than 25 years since its identification, a new wave of interest on Stat3 has emerged within the last decade after the discovery of the novel finding that Stat3 is also present in mitochondria (mitochondrial Stat3), where it can regulate the mitochondrial respiratory chain [11]. This discovery was made by Larner and Levy’s groups and was initially received with some skepticism. However, over the last few years, the interest and work surrounding mitochondrial Stat3 (mitoStat3) has been greatly expanded primarily in cancer, cardiology, and neuroscience. Surprisingly, mitoStat3 has not yet fully launched within the immunology field, where only a few reports about mitoStat3 exist. The goals of this perspective are to (1) briefly summarize what we know about mitoStat3 (recently reviewed by others), (2) review what we know about Stat3 and mitoStat3 in T and B cells, (3) discuss how some of the functions assigned to Stat3 as a transcriptional factor in the immune system could be indeed mediated by mitoStat3, and (4) encourage immunologists to study mitoStat3 in depth in lymphocytes primarily with the emerging interest in immunometabolism. Jak inhibitors are currently being tested in inflammatory diseases, but mitoStat3 has not been considered as a potential target for inflammatory diseases. Increasing knowledge about mitoStat3 regulation and function may help to develop a new generation of Stat3 inhibitors as therapeutics.

## 2. Mitochondrial Stat3 as a Modulator of Mitochondria Function

Initial observations suggesting that Stat3 could have a role in mitochondria came from a report indicating that Stat3 can bind GRIM-19, which is considered a component of Complex I of the electron transport chain (ETC) [12]. A few years later, two studies reported that Stat3 can also localize in mitochondria, where it associates with Complex I of the ETC and promotes Complex I activity and mitochondrial respiration [11,13]. In addition, association with Complex II or Complex V (ATP synthase) has also been reported but is less clear [11,13,14,15]. mitoStat3 also impacts the ATP production [16,17]. The absence of mitoStat3 reduces the levels of ATP in Ras-transformed cells [11]. The selective depletion of Stat3 also decreases ATP production in astrocytes [15].Thus, in the majority of tissues, the production of ATP is increased by mitoStat3 (Figure 1). However, we reported that in mouse CD4 T cells activated with IL-6, the mitoStat3 translocation does not change ATP levels, although in these cells mitoStat3 modulates mitochondrial Ca^2+^ content [16].

In addition to its association with ETC Complexes, more recent studies have shown that mitoStat3 also binds to cyclophilin D (CypD), and this association inhibits the opening of the mitochondrial permeability transition pore (MPTP), thereby reducing mitochondrial reactive oxygen species (ROS) production [18,19]. Stat3 has also been proposed to reduce ROS production by facilitating the formation of Respiratory Supecomplexes in the mitochondria and minimizing electron leak during the transport in the ETC [16]. While most studies support a role of mitoStat3 as a ROS reducing factor, others have reported increased ROS production by mitoStat3 in cancer cells [16,19,20], but the mechanisms have not been elucidated or proposed.

Tyr^705^ phosphorylation occurs first and is necessary for STAT3 dimerization and DNA binding, while the Ser^727^ phosphorylation is a secondary event required for enhancing transcriptional activity of Stat3 [4]. Although both Tyr^705^ and Ser^727^ phosphorylation of Stat3 are present in mitochondria, Ser^727^ seems to be more critical for regulation of ETC activity by mitoStat3 [11,13]. The mechanisms involved in Stat3 import into mitochondria are still not completely understood. Reports have shown that GRIM-19 acts as a chaperone recruiting Stat3 into the mitochondrial inner membrane complexes [21]. The mitochondrial importer Tom20 also participate in Stat3 entry into mitochondria [18,22]. A recent study has shown that mitochondrial translocation of Stat3 is promoted by its acetylation, but it remains unclear whether this protein modification affects the association of Stat3 with GRIM-19 or Tom20 [23].

Most studies investigating the relevance of mitoStat3 have been focused on its role on cancer biology, cardiovascular diseases and neurological diseases. mitoStat3 has a cardio protector effect against ischemia/reperfusion injury by inhibiting MPTP opening, preserving the mitochondrial respiratory function, and minimizing ROS generation [18,24]. In cancer, mitoStat3 is critical for survival of ras-transformed mouse embryonic cells [11]. The participation of mitoStat3 was also shown in tumors such as chronic lymphoid leukemia and pancreatic and breast cancers [20,22,25]. Recently it has been demonstrated that an inhibitor of Stat3 interferes with mitochondrial activity, inducing a synthetic lethality effect in glucose-depleted cancer cells [26]. Together, these data suggest that selective inhibition of mitoStat3 could be a target for cancer treatment. mitoStat3 has also been shown to play a role in neuronal cells where it induces neurite outgrowth in response to nerve growth factor (NGF) [27,28] and increases axon regrowth [29]. Moreover, mitoStat3 is upregulated following spinal cord injury [30]. Thus, the presence and function of Stat3 in mitochondria is not restricted to malignant cells but most likely is determined by the metabolic needs of the cells.

## 3. Stat3 as a Modulator of Gene Transcription in T and B Lymphocytes

Stat3 is emerging as a key modulator of the immune response due to its role in the regulation of and the regulation by key cytokines (e.g., IL-6, IL-21) that shape both the T cell response as well as the B cell response. In addition to studies using cell-specific Stat3 deficient mice, the relevance of Stat3 in shaping the immune response has been further demonstrated by genetic human immunodeficiency and immune dysregulations caused by mutations in the Stat3 gene [31,32]. Loss-of-function mutations in Stat3 affecting predominantly the DNA binding and the transactivation domain cause autosomal dominant hyper IgE syndrome (AD-HIES), a primary immunodeficiency characterized by defects in T and B lymphocytes [31,33]. Patients with AD-HIES have elevated levels of IgE, lower levels of IgG, and recurrent opportunistic infections (e.g., pneumonia, candidiasis) [34]. Interestingly, gain of function (GOF) mutations of Stat3 in humans can result in early stages of autoimmunity (e.g., type I diabetes, arthritis) but can also cause some types of immunodeficiencies such as hypogammaglobulinemia, as well as increased recurrent infections [32,35], but the underlined mechanisms for this effect remain unclear.

### 3.1. Stat3 in CD4 Cell Function

Upon activation through T cell receptor (TCR) and co-stimulation signals, naïve CD4 cells undergo proliferation and differentiation into effector T helper (Th) cells that can produce specific cytokines. Importantly, the cytokine environment is the main factor that determines which the type of Th subset naïve cells differentiate during activation. Activation of Stat3 as a transcription factor by this cytokine environment (primarily IL-6) makes Stat3 an important master regulator CD4 cell differentiation and effector function. Effector Th17 cells produce IL-17 and are generated predominantly when naïve CD4 cells are activated in the presence of TGFβ and IL-6 (although IL-6 does not promote Th17 differentiation by itself) [36]. Because of the need for IL-6, differentiation of Th17 cells is dependent on Stat3. However, regulation of IL-17 expression by Stat3 is in indirect effect of Stat3 promoting the expression of RORγ and RORα transcriptional factors that then mediate the expression of IL-17 gene [37,38]. In agreement with the contribution of Stat3 to Th17 cell differentiation, IL-17 production by CD4 Th17 cells is impaired in AD-HIES patients [39]. The recurrent *Candida* spp. infections in AD-HIES patients also correlate with an impaired Th17 response since IL-17 is crucial for controlling fungal infections [40]. Intriguingly, however, no increase in IL-17 production is observed in patients with Stat3 gain of function (GOF) mutations, suggesting that activation of Stat3 as a transcription factor is not sufficient to promote IL-17 expression. It is possible that the “nuclear function” (e.g., mediating transcription) is enhanced in the GOF mutant Stat3, but “unconventional functions” of Stat3 (e.g., mitochondrial Stat3) could be impaired, and these unconventional functions could also contribute to the differentiation of Th17 cells (see below). 

Differentiation of naïve CD4 cells into effector Th1 cells is mostly mediated by IL-12 through the activation of Stat4. Th1 cells produce primarily IFNγ [41]. IFNγ production is increased in Stat3-deficient Th1 cells [42]. We have shown that IL-6 inhibits Th1 differentiation by activating Stat3 and Stat3 mediating SOCS1 expression [43]. Increased SOCS levels interfere with activation of Stat1 by IFNγ, a signaling pathway required for full Th1 differentiation [43]. Thus, Stat3 is a negative regulator of Th1 differentiation. Similarly, Stat3 seems to be a negative regulator for the generation of T regulatory (Treg) cells [44].

CD4 Th2 cells produce IL-4, IL-13, and IL-5, and Th2 differentiation of naïve CD4 cells is predominantly promoted by IL-4 through Stat6, with no requirement of Stat3 as a transcription factor. We have shown that IL-6 also promotes the differentiation of naïve CD4 cells into effector Th2 cells that produce IL-4 and IL-13 but not IL-5 [45]. IL-6 promotes Th2 differentiation by inducing IL-4 production early during activation. Secreted IL-4 then provides further positive feedback to fully differentiate these cells into Th2 cells [45]. The effect of IL-6 on IL-4 gene expression is mediated by the nuclear factor of activated T cells (NFAT) transcription factor [46]. Interestingly, although Stat3 as a transcription factor is not required for IL-4 induction by IL-6, mitoStat3 indirectly contributes to a prolonged IL-4 production by IL-6 through its effect on mitochondrial Ca^2+^ (see below for more detail) [16]. 

CD4 T follicular helper (Tfh) cells are effector cells that localize primarily in the follicles where they provide help to B cells by producing IL-21 and promote isotype switching and survival of plasma B cells [47,48]. In mouse, IL-6 via Stat3 is the major cytokine to promote Tfh cell differentiation, while in human other Stat3-activating cytokines such as IL-12 and IL27 also contribute [49]. We and others have shown that IL-6 is the most efficient cytokine to promote IL-21 production by naïve CD4 cells both in mouse and human, with low dose of IL-6 being sufficient [50,51]. The main effect of Stat3 as transcription factor is not through a direct effect on IL-21 gene promoter, but by inducing the expression of Bcl6, and Bcl6 inducing IL-21 gene expression [52]. In addition to its conventional function as a transcription factor, we have shown that mitoStat3 also contributes to sustain IL-21 production by CD4 cells in the presence of IL-6 by regulating mitochondrial Ca^2+^.

### 3.2. Stat3 in CD8 Cell Function

Similar to CD4 cells, naïve CD8 cells also differentiate into effector cells upon activation through TCR. However, unlike the helper function of effector CD4 cells, the major function of effector CD8 cells is cytotoxicity via the secretion of granzyme (proteases) and perforin. Perforin forms pores in the target cells (e.g., infected cells) to allow granzyme to enter and cause cell death. CD8 cells are also an important source of IFNγ. Most of the studies on Stat3 in CD8 cells have been associated with IL-21 [53]. IL-21 does not seem to have significant effect on naïve CD8 cells activation and proliferation, including in CD8 cells from AD-HIES patients [54]. IL-21 contributes to the generation of long-lived memory CD8 cells and AD-HIES patients are more susceptible to viral reactivation, correlating with impaired memory CD8 cells [54]. While cytotoxicity is the main function of effector cells, we have recently shown that the presence of IL-6 during the activation of CD8 cells induces the production of IL-21 and effector CD8 cells become helpers of B cells [55]. Stat3 is required for the induction of IL-21 gene expression and production in CD8 cells by IL-6 [55]. IL-6-activated CD8 cells provide IL-21 to B cells and thereby promote IgG production by B cells [55]. During influenza virus infection, IL-6 is required for CD8 cells to produce IL-21 in the lung and contributes to in vivo antibody response. Thus, Stat3 mediates a new unexpected function of CD8 cells. The increased susceptibility of AD-HIES patients to recurrence of viral infection could be due to an impairment of CD8 function as B cell helpers.

### 3.3. Stat3 in B Cell Function

The main function of B cells is the production of antibodies as a mechanism of defense. Upon activation of B cells through the B cell receptor, these cells become plasma B cells and start to secrete antibodies. First, activated B cells secrete IgM, but in the presence of cytokines they undergo isotype switching and start producing either IgG (IgG1, IgG2, IgG3, and IgG4 in humans), IgA, IgD, or IgE. Generation of memory B cells is also dependent on cytokines. IL-21 is known to play a key role in isotype switching as well as in survival of memory and plasma B cells [56]. Studies in conditional knockout mice have shown that Stat3 in B cells is required for the differentiation of IgG secreting plasma B cells in part by inducing the expression of Blimp-1, a transcription factor promoting B cell maturation [57,58]. Similarly, naïve B cells from AD-HIES patients fail to differentiate into antibody-secreting cells when activated with CD40L and IL-21 [59,60], indicating that Stat3 is also required for B cell production of antibodies in human. Interestingly, unlike the studies in mice, isotype switching to IgG in activated B cells from Stat3 mutant patients does not seem to be impaired [60]. However, it is possible that analysis of total IgG may have mask effects of Stat3 on specific IgG isotypes. Thus, we have shown that IL-21 induces IgG4 production by activated human B cells from healthy volunteers, while IL-4 promotes switching to other isotypes but not to IgG4 [61]. The contribution of Stat3 in isotype switching to IgG4 has not yet been investigated. Stat3 also contributes to survival and expansion of memory B cells [59]. Although only a few memory B cells can be detected in AD-HIES patients, these remaining cells seem to be capable of differentiating into antibody-secreting cells in response to IL-21 despite the impaired transcription function of Stat3. This phenotype contrasts with the inability of B cells from Stat3 deficient mice to produce antibodies. As we discuss in the next section, it is possible that secretion of antibody may require an unconventional function of Stat3, independent of its role as transcription factor, that is retained in the mutant Stat3 B cells from AD-HIES patients.

## 4. Mitochondrial Stat3 in T and B Cells: What We Know and What We Need to Know

With the well-established function of Stat3 as a transcriptional factor, incorporating a novel function of Stat3 in the research field has been a challenge. Thus, following the initial studies reporting that Stat3 is present in mitochondria and regulates the electron transport chain, several years of research were necessary to broaden mitoStat3 research. Within the last few years, a number of studies have reported the presence of mitoStat3 in cancer cells and have described its role in cancer progression [26]. mitoStat3 has also been explored extensively the neuroscience field, in part due to the key role that mitochondria has in the central nervous system [29]. Interestingly, despite the number of studies on Stat3 in lymphocytes and other components of the immune response, there is little known about the regulation and the role of mitoStat3 in the immune system.

Following the initial observations describing the presence of Stat3 in mitochondria and considering the major role that IL-6 plays on CD4 cells, we decided to investigate whether Stat3 was present in mitochondria in these cells. We have shown marginal levels of Stat3 in mitochondria in CD4 cells prior to activation, but upon activation in the presence of IL-6, Stat3 accumulates in mitochondria [16]. The levels of Stat3 in mitochondria are comparable to those in the nucleus of CD4 cells activated with IL-6. According to the previously described effects of Stat3 on mitochondrial membrane potential (MMP) [11,13], we have shown that IL-6 sustains higher MMP during activation of CD4 cells and this effect is mediated by Stat3 [16]. Although some studies have described that Stat3 can also promote the generation of ROS due to its effect of increasing Complex I activity and MMP, ROS production is decreased in CD4 cells activated with IL-6 [16]. The formation of mitochondrial respiratory supercomplexes (RCS) [62,63,64], formed primarily of Complex I together with Complex III and Complex IV, is now well established and their structure in mammalian cells has been recently described [65,66,67]. The function of RSC is to facilitate the transfer of electrons between Complexes within the ETC by bringing them together and reducing electron leak (and ROS), as well as to enhance Complex I activity [68,69,70,71,72]. We have shown that IL-6 promotes the formation of supercomplexes [16]. Stat3 associates with monomeric Complex I, most likely through GRIM19 [21]. Interestingly, we have shown that Stat3 is also present in respiratory supercomplexes in CD4 cells activated in the presence of IL-6. Follow up studies are needed to investigate the role of Stat3 in the formation of supercomplexes, activity of Complex I in the supercomplexes, and the impact on ROS production.

Increased ETC activity and MMP by mitoStat3 has been linked to increased synthesis of mitochondrial ATP and increased OXPHOS [13]. In CD4 cells, the increased MMP triggered by IL-6 through Stat3 is uncoupled from OXPHOS. Instead, increased MMP is used to promote mitochondrial Ca^2+^ accumulation, another function of mitochondrial membrane potential. Mitochondrial Ca^2+^ then regulates the basal levels of cytosolic Ca^2+^ [16]. Stat3 deficiency causes a reduction in mitochondrial Ca^2+^ [16]. Thus, through its effect on ETC mitoStat3 can regulate both mitochondrial ATP production (OXPHOS) and mitochondrial Ca^2+^ homeostasis.

**Figure 2:**
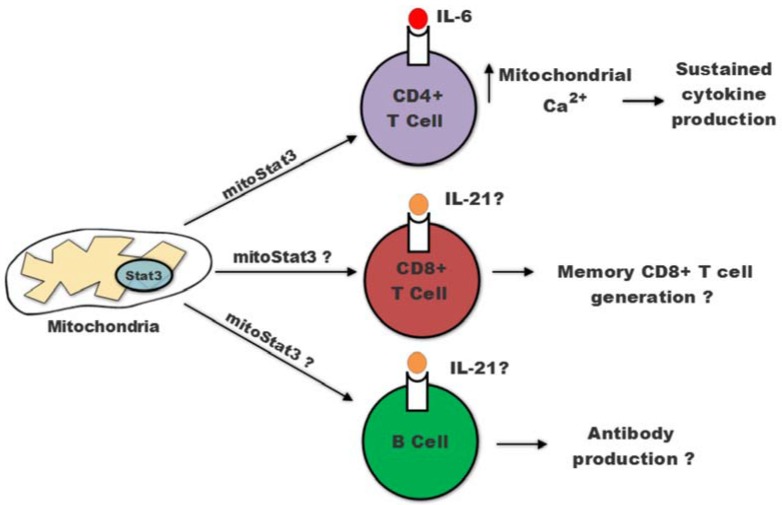
mitoStat3 function in lymphocyte. CD4 cells activated with IL-6 increase mitochondrial Ca^2+^ via mitoStat3 and contributes to a sustained expression of cytokines. mitoStat3 may participates in CD8 cell memory generation by IL21. In B cells, the production of antibody mediated by IL-21 may requires mitoStat3.

Although no other descriptions of mitoStat3 have been reported for T and B cells, a number of functions in these cells assigned to Stat3 as a transcription factor could be mediated through mitoStat3. In addition, findings that could not be explained based on the conventional function of Stat3 as transcription factor may be explained by the function of mitoStat3. This is the case for some of the phenotypic characteristics found in patients with gain of function (GOF) mutation in Stat3 where there is an accumulation of Stat3 in the nucleus [32]. For instance, while a persistence immune response and autoimmunity would be expected, patients with GOF Stat3 mutations often develop immunodeficiencies and are more susceptible to infections. Mitochondrial Stat3 has not been investigated in these patients in either T or B cells, but it is possible that the enhanced nuclear accumulation of Stat3 causes a reduction in the levels of mitochondrial Stat3. Thus, functions mediated by mitoStat3 could be compromised in the lymphocytes of these patients. In this regard, we have shown that IL-6-mediated mitochondrial Ca^2+^ sustains the production of the two cytokines (IL-21 and IL-4) known to be regulated by IL-6 in CD4 cells [16] (Figure 2), and another study has shown that the production of IL-21 and IL-4 by Tfh cells in germinal centers is dependent on Ca^2+^ [73]. It is therefore possible that mitoStat3 contributes to sustain prolonged cytokine production in CD4 cells in inflammatory diseases.

Since Stat3 as a transcription factor promotes B cell differentiation and antibody production [59,60], B cells from GOF Stat3 mutant patients would be predicted to be superior in producing antibodies. However, in contrast to this prediction, some patients develop hypoglobulinemia and have reduced isotype switching by memory B cells [60]. This phenotype could be explained if mitoStat3 contributes to B cell antibody production and the levels of mitoStat3 in GOF B cells are reduced. Interestingly, although the number of memory B cells is reduced in AD-HIES patients with a loss of function (LOF) Stat3 mutation, the remaining memory B cells can respond to IL-21 and secrete antibodies normally [60]. While these LOF Stat3 mutants fail to mediate transcription, they still can translocate to mitochondria and mediate the unconventional functions. It is therefore possible that the remaining mitoStat3 in those AD-HIES memory B cells is sufficient to contribute to antibody production in response to IL-21 (Figure 2). Secretion is a process highly dependent on ATP and Ca^2+^ [74]. Although it has not yet been investigated, mitochondria could be the major source of both ATP and Ca^2+^ for secretion of antibodies by plasma cells, and mitoStat3 could contribute to this process as well (Figure 2). In summary, while no studies have investigated the presence of Stat3 in mitochondria and the potential function of mitoStat3 in B cells, future studies should be warranted. 

As mentioned above, although Stat3 does not contribute to proliferation of effector CD8 cells, a number of studies have shown the role of Stat3 in memory CD8 cells both in mouse and human. No studies have reported mitochondrial Stat3 in CD8 cells, However, it is well established that memory CD8 cells are more dependent on mitochondrial respiration as a source of energy instead of glycolysis, the common mechanism used by effector CD8 cells during proliferation [75]. Memory CD8 cells have been shown to use fatty acid oxidation in mitochondria [76]. Thus, mitochondrial function is clearly more relevant for memory CD8 cells than effector cells. Considering the effect of Stat3 in promoting Complex I activity and mitochondrial ATP synthesis, one would think that mitoStat3 could play a role in maintaining mitochondrial respiration in these cells (Figure 2). As mentioned above, IL-21 is known to promote the generation of long-lived memory CD8 cells [77,78]. This effect could be by maintaining mitochondrial metabolism through mitoStat3. Investigation in these areas could bring a new perspective for Stat3 in CD8 cells.

## 5. Need for an Unconventional Design Thinking Involving mitoStat3 for Novel Therapies in Inflammatory Diseases

According to the function of Stat3 as a transcriptional factor—and its role in cell survival and angiogenesis—a number of DNA-based Stat3 inhibitors have been generated, and some have been tested in clinical trials for advanced cancer [17,79,80]. The nature of those inhibitors is broad. Antisense oligonucleotides to inhibit Stat3 expression (e.g., AZD9150) have been used in several clinical trials for diffuse large B cell lymphoma, metastatic HCC, malignant ascites, advanced solid tumors, and non-small-cell lung cancer [80,81,82]. Double-stranded oligonucleotides containing the Stat3 binding site have also been proposed as inhibitors in preclinical studies for cancer [83]. Several peptide-based Stat3 inhibitors have been used in mouse models of cancer but not in Phase I or 2 clinical trials [84,85]. Most Stat3 blockers tested clinically in cancer are small molecules. C188-9 is a small molecule inhibitor of Stat3 provided orally that is currently in an ongoing Phase I clinical trial for patients with advanced cancers [86], but results have yet to be released. OPB-51602 is a small molecule that inhibits Stat3 phosphorylation without affecting the Jak kinases [87]. During Phase I trials in refractory cancer patients, OPB-51602 was well tolerated and showed some anti-tumor activity. However, long-term administration had some toxic effects [87,88]. A number of other inhibitors of Stat3 have been tested in mouse models but not yet in clinical trials. Most of the inhibitors do not have an effect on mitochondrial Stat3, which has emerged as an alternative target within the discovery programs of a number of pharmaceuticals. MDC-1112, phospho-valproic acid, was identified as a potential inhibitor of mitoStat3 because it reduces mitochondrial Stat3 accumulation [22]. Although animal models have shown efficacy of this inhibitor in pancreatic cancer and other solid tumors [22], no clinical trials have been yet initiated. 

Interestingly, despite the strong evidence from in vitro studies and animal models indicating that Stat3 could be a target for chronic inflammatory disease, no clinical trials have been initiated for long-term treatments. STA-21, another small molecule Stat3 inhibitor, is the only known inhibitor that has been tested in an inflammatory disease, but the administration for psoriasis was topical and only for two weeks [89]. Nevertheless, the lack of ongoing clinical trials does not reflect a lack of interest on Stat3 as a potential target in inflammatory diseases. Most likely this is due to the fact that IL-6, one of the major inducers of Stat3, has been a successful target for the treatment of rheumatoid arthritis and other autoimmune diseases through the use of biological therapies (e.g., tocilizumab) [90].

However, biological therapies come with important limitations that prevent their use broadly and worldwide. As any other biological therapy, treatment with tocilizumab (anti-IL-6R blocker) is costly and does not cure inflammatory diseases, meaning often the treatment is maintained for life. The average cost for tocilizumab is about $2000 per infusion, and it is commonly required to be taken once per month. In addition, treatment with tocilizumab, like most other biological therapies, requires a visit to the clinic where the infusions of the antibody are provided, clearly something that not every patient can manage because of the time and the accessibility to the appropriate clinics. Thus, while blocking IL-6R or IL-6 is an optional therapy for some patients with specific inflammatory diseases, it is not an option for all. Because of these limitations, pharmaceuticals and the health system are still investigating for more affordable treatments. Indeed, a number of JAK inhibitors are being tested in clinical trials [91]. These inhibitors are not specific for Stat3 since they affect several Stat molecules and multiple pathways. As a result, complications following long-term treatments in patients with inflammatory diseases with JAK inhibitors have been a challenge to overcome. To date, only Xeijanz JAK inhibitor has been approved by the FDA for treatment of Rheumatoid Arthritis, while no inhibitors have yet been approved in Europe [92]. Thus, targeting Stat3 in chronic inflammatory diseases is still an option that will need to be tested. Considering the emerging interest on mitochondrial Stat3, as well as the growing interest regarding mitochondria in regulating immune response, targeting mitochondrial Stat3 in inflammatory diseases could be a novel strategy that may exhibit less challenges. Inhibitors targeting predominantly mitochondrial Stat3 instead of Stat3-mediated transcription may also have less side effect since Stat3 as transcription factor has multiple gene targets. Although no studies have yet investigated mitochondrial Stat3 in human T or B cells, our recent (unpublished) studies suggest that IL-6 also regulates mitochondrial function in T cells. Future studies will be needed to explore the regulation of mitoStat3 in CD4 cells and B cells in healthy conditions as well as in chronic inflammatory diseases.

## References

[1] Lutticken C., Wegenka U.M., Yuan J., Buschmann J., Schindler C., Ziemiecki A., Harpur A.G., Wilks A.F., Yasukawa K., Taga T. (1994). Association of transcription factor APRF and protein kinase Jak1 with the interleukin-6 signal transducer gp130. Science.

[2] Zhong Z., Wen Z., Darnell J.E. (1994). Stat3: A STAT family member activated by tyrosine phosphorylation in response to epidermal growth factor and interleukin-6. Science.

[3] Delgoffe G.M., Vignali D.A. (2013). STAT heterodimers in immunity: A mixed message or a unique signal?. Jakstat.

[4] Wen Z., Zhong Z., Darnell J.E. (1995). Maximal activation of transcription by Stat1 and Stat3 requires both tyrosine and serine phosphorylation. Cell.

[5] Rose-John S. (2018). Interleukin-6 Family Cytokines. Cold Spring Harb. Perspect. Biol..

[6] Niemand C., Nimmesgern A., Haan S., Fischer P., Schaper F., Rossaint R., Heinrich P.C., Muller-Newen G. (2003). Activation of STAT3 by IL-6 and IL-10 in primary human macrophages is differentially modulated by suppressor of cytokine signaling 3. J. Immunol..

[7] Murray P.J. (2006). Understanding and exploiting the endogenous interleukin-10/STAT3-mediated anti-inflammatory response. Curr. Opin. Pharmacol..

[8] Raeber M.E., Zurbuchen Y., Impellizzieri D., Boyman O. (2018). The role of cytokines in T-cell memory in health and disease. Immunol. Rev..

[9] Ward L.D., Howlett G.J., Discolo G., Yasukawa K., Hammacher A., Moritz R.L., Simpson R.J. (1994). High affinity interleukin-6 receptor is a hexameric complex consisting of two molecules each of interleukin-6, interleukin-6 receptor, and gp-130. J. Biol. Chem..

[10] Silver J.S., Hunter C.A. (2010). gp130 at the nexus of inflammation, autoimmunity, and cancer. J. Leukoc. Biol..

[11] Gough D.J., Corlett A., Schlessinger K., Wegrzyn J., Larner A.C., Levy D.E. (2009). Mitochondrial STAT3 supports Ras-dependent oncogenic transformation. Science.

[12] Lufei C., Ma J., Huang G., Zhang T., Novotny-Diermayr V., Ong C.T., Cao X. (2003). GRIM-19, a death-regulatory gene product, suppresses Stat3 activity via functional interaction. EMBO J..

[13] Wegrzyn J., Potla R., Chwae Y.J., Sepuri N.B., Zhang Q., Koeck T., Derecka M., Szczepanek K., Szelag M., Gornicka A. (2009). Function of mitochondrial Stat3 in cellular respiration. Science.

[14] Demaria M., Giorgi C., Lebiedzinska M., Esposito G., D’Angeli L., Bartoli A., Gough D.J., Turkson J., Levy D.E., Watson C.J. (2010). A STAT3-mediated metabolic switch is involved in tumour transformation and STAT3 addiction. Aging.

[15] Sarafian T.A., Montes C., Imura T., Qi J., Coppola G., Geschwind D.H., Sofroniew M.V. (2010). Disruption of astrocyte STAT3 signaling decreases mitochondrial function and increases oxidative stress in vitro. PLoS ONE.

[16] Yang R., Lirussi D., Thornton T.M., Jelley-Gibbs D.M., Diehl S.A., Case L.K., Madesh M., Taatjes D.J., Teuscher C., Haynes L. (2015). Mitochondrial Ca^2+^ and membrane potential, an alternative pathway for interleukin 6 to regulate CD4 cell effector function. Elife.

[17] Yang R., Rincon M. (2016). Mitochondrial Stat3, the need for design thinking. Int. J. Biol. Sci..

[18] Boengler K., Hilfiker-Kleiner D., Heusch G., Schulz R. (2010). Inhibition of permeability transition pore opening by mitochondrial STAT3 and its role in myocardial ischemia/reperfusion. Basic Res. Cardiol..

[19] Meier J.A., Hyun M., Cantwell M., Raza A., Mertens C., Raje V., Sisler J., Tracy E., Torres-Odio S., Gispert S. (2017). Stress-induced dynamic regulation of mitochondrial STAT3 and its association with cyclophilin D reduce mitochondrial ROS production. Sci. Signal..

[20] Zhang Q., Raje V., Yakovlev V.A., Yacoub A., Szczepanek K., Meier J., Derecka M., Chen Q., Hu Y., Sisler J. (2013). Mitochondrial localized Stat3 promotes breast cancer growth via phosphorylation of serine 727. J. Biol. Chem..

[21] Tammineni P., Anugula C., Mohammed F., Anjaneyulu M., Larner A.C., Sepuri N.B. (2013). The import of the transcription factor STAT3 into mitochondria depends on GRIM-19, a component of the electron transport chain. J. Biol. Chem..

[22] Mackenzie G.G., Huang L., Alston N., Ouyang N., Vrankova K., Mattheolabakis G., Constantinides P.P., Rigas B. (2013). Targeting mitochondrial STAT3 with the novel phospho-valproic acid (MDC-1112) inhibits pancreatic cancer growth in mice. PLoS ONE.

[23] Xu Y.S., Liang J.J., Wang Y., Zhao X.J., Xu L., Xu Y.Y., Zou Q.C., Zhang J.M., Tu C.E., Cui Y.G. (2016). STAT3 undergoes acetylation-dependent mitochondrial translocation to regulate pyruvate metabolism. Sci. Rep..

[24] Zhang G., Sheng M., Wang J., Teng T., Sun Y., Yang Q., Xu Z. (2018). Zinc improves mitochondrial respiratory function and prevents mitochondrial ROS generation at reperfusion by phosphorylating STAT3 at Ser(727). J. Mol. Cell. Cardiol..

[25] Capron C., Jondeau K., Casetti L., Jalbert V., Costa C., Verhoeyen E., Masse J.M., Coppo P., Bene M.C., Bourdoncle P. (2014). Viability and stress protection of chronic lymphoid leukemia cells involves overactivation of mitochondrial phosphoSTAT3Ser_727_. Cell Death Dis..

[26] Genini D., Brambilla L., Laurini E., Merulla J., Civenni G., Pandit S., D’Antuono R., Perez L., Levy D.E., Pricl S. (2017). Mitochondrial dysfunction induced by a SH2 domain-targeting STAT3 inhibitor leads to metabolic synthetic lethality in cancer cells. Proc. Natl. Acad. Sci. USA.

[27] Zhou L., Too H.P. (2011). Mitochondrial localized STAT3 is involved in ngf induced neurite outgrowth. PLoS ONE.

[28] Zhou L., Too H.P. (2013). Gdnf family ligand dependent STAT3 activation is mediated by specific alternatively spliced isoforms of GFRalpha2 and RET. Biochim. Biophys. Acta.

[29] Luo X., Ribeiro M., Bray E.R., Lee D.H., Yungher B.J., Mehta S.T., Thakor K.A., Diaz F., Lee J.K., Moraes C.T. (2016). Enhanced transcriptional activity and mitochondrial localization of STAT3 co-induce axon regrowth in the adult central nervous system. Cell Rep..

[30] Park K.W., Lin C.Y., Benveniste E.N., Lee Y.S. (2016). Mitochondrial STAT3 is negatively regulated by SOCS3 and upregulated after spinal cord injury. Exp. Neurol..

[31] Holland S.M., DeLeo F.R., Elloumi H.Z., Hsu A.P., Uzel G., Brodsky N., Freeman A.F., Demidowich A., Davis J., Turner M.L. (2007). STAT3 mutations in the hyper-IgE syndrome. N. Engl. J. Med..

[32] Flanagan S.E., Haapaniemi E., Russell M.A., Caswell R., Allen H.L., De Franco E., McDonald T.J., Rajala H., Ramelius A., Barton J. (2014). Activating germline mutations in STAT3 cause early-onset multi-organ autoimmune disease. Nat. Genet..

[33] Minegishi Y., Saito M., Tsuchiya S., Tsuge I., Takada H., Hara T., Kawamura N., Ariga T., Pasic S., Stojkovic O. (2007). Dominant-negative mutations in the DNA-binding domain of STAT3 cause hyper-IgE syndrome. Nature.

[34] Freeman A.F., Holland S.M. (2008). The hyper-IgE syndromes. Immunol. Allergy Clin. N. Am..

[35] Haapaniemi E.M., Kaustio M., Rajala H.L., van Adrichem A.J., Kainulainen L., Glumoff V., Doffinger R., Kuusanmaki H., Heiskanen-Kosma T., Trotta L. (2015). Autoimmunity, hypogammaglobulinemia, lymphoproliferation, and mycobacterial disease in patients with activating mutations in STAT3. Blood.

[36] Basso A.S., Cheroutre H., Mucida D. (2009). More stories on Th17 cells. Cell Res..

[37] Zhou L., Ivanov I.I., Spolski R., Min R., Shenderov K., Egawa T., Levy D.E., Leonard W.J., Littman D.R. (2007). IL-6 programs T(H)-17 cell differentiation by promoting sequential engagement of the IL-21 and IL-23 pathways. Nat. Immunol..

[38] Yang X.O., Panopoulos A.D., Nurieva R., Chang S.H., Wang D., Watowich S.S., Dong C. (2007). STAT3 regulates cytokine-mediated generation of inflammatory helper T cells. J. Biol. Chem..

[39] Ma C.S., Chew G.Y., Simpson N., Priyadarshi A., Wong M., Grimbacher B., Fulcher D.A., Tangye S.G., Cook M.C. (2008). Deficiency of Th17 cells in hyper IgE syndrome due to mutations in STAT3. J. Exp. Med..

[40] Cypowyj S., Picard C., Marodi L., Casanova J.L., Puel A. (2012). Immunity to infection in IL-17-deficient mice and humans. Eur. J. Immunol..

[41] O’Shea J.J., Paul W.E. (2010). Mechanisms underlying lineage commitment and plasticity of helper CD4+ T cells. Science.

[42] Wan C.K., Andraski A.B., Spolski R., Li P., Kazemian M., Oh J., Samsel L., Swanson P.A., McGavern D.B., Sampaio E.P. (2015). Opposing roles of STAT1 and STAT3 in IL-21 function in CD4+ T cells. Proc. Natl. Acad. Sci. USA.

[43] Diehl S., Anguita J., Hoffmeyer A., Zapton T., Ihle J.N., Fikrig E., Rincon M. (2000). Inhibition of Th1 differentiation by IL-6 is mediated by SOCS1. Immunity.

[44] Nishihara M., Ogura H., Ueda N., Tsuruoka M., Kitabayashi C., Tsuji F., Aono H., Ishihara K., Huseby E., Betz U.A. (2007). IL-6-gp130-STAT3 in T cells directs the development of IL-17+ Th with a minimum effect on that of Treg in the steady state. Int. Immunol..

[45] Rincon M., Anguita J., Nakamura T., Fikrig E., Flavell R.A. (1997). Interleukin (IL)-6 directs the differentiation of IL-4-producing CD4+ T cells. J. Exp. Med..

[46] Diehl S., Chow C.W., Weiss L., Palmetshofer A., Twardzik T., Rounds L., Serfling E., Davis R.J., Anguita J., Rincon M. (2002). Induction of NFATc2 expression by interleukin 6 promotes t helper type 2 differentiation. J. Exp. Med..

[47] Bryant V.L., Ma C.S., Avery D.T., Li Y., Good K.L., Corcoran L.M., de Waal Malefyt R., Tangye S.G. (2007). Cytokine-mediated regulation of human b cell differentiation into Ig-secreting cells: Predominant role of IL-21 produced by CXCR5+ T follicular helper cells. J. Immunol..

[48] Linterman M.A., Beaton L., Yu D., Ramiscal R.R., Srivastava M., Hogan J.J., Verma N.K., Smyth M.J., Rigby R.J., Vinuesa C.G. (2010). IL-21 acts directly on B cells to regulate Bcl-6 expression and germinal center responses. J. Exp. Med..

[49] Batten M., Ramamoorthi N., Kljavin N.M., Ma C.S., Cox J.H., Dengler H.S., Danilenko D.M., Caplazi P., Wong M., Fulcher D.A. (2010). IL-27 supports germinal center function by enhancing IL-21 production and the function of T follicular helper cells. J. Exp. Med..

[50] Dienz O., Eaton S.M., Bond J.P., Neveu W., Moquin D., Noubade R., Briso E.M., Charland C., Leonard W.J., Ciliberto G. (2009). The induction of antibody production by IL-6 is indirectly mediated by IL-21 produced by CD4+ T cells. J. Exp. Med..

[51] Diehl S.A., Schmidlin H., Nagasawa M., Blom B., Spits H. (2012). IL-6 triggers IL-21 production by human CD4+ T cells to drive STAT3-dependent plasma cell differentiation in B cells. Immunol. Cell Biol..

[52] Nurieva R.I., Chung Y., Hwang D., Yang X.O., Kang H.S., Ma L., Wang Y.H., Watowich S.S., Jetten A.M., Tian Q. (2008). Generation of T follicular helper cells is mediated by interleukin-21 but independent of T helper 1, 2, or 17 cell lineages. Immunity.

[53] Leonard W.J., Wan C.K. (2016). IL-21 signaling in immunity. F1000Res..

[54] Siegel A.M., Heimall J., Freeman A.F., Hsu A.P., Brittain E., Brenchley J.M., Douek D.C., Fahle G.H., Cohen J.I., Holland S.M. (2011). A critical role for STAT3 transcription factor signaling in the development and maintenance of human t cell memory. Immunity.

[55] Yang R., Masters A.R., Fortner K.A., Champagne D.P., Yanguas-Casas N., Silberger D.J., Weaver C.T., Haynes L., Rincon M. (2016). IL-6 promotes the differentiation of a subset of naive CD8+ T cells into IL-21-producing B helper CD8+ T cells. J. Exp. Med..

[56] Wu Y., van Besouw N.M., Shi Y., Hoogduijn M.J., Wang L., Baan C.C. (2016). The biological effects of IL-21 signaling on B-cell-mediated responses in organ transplantation. Front. Immunol..

[57] Fornek J.L., Tygrett L.T., Waldschmidt T.J., Poli V., Rickert R.C., Kansas G.S. (2006). Critical role for Stat3 in T-dependent terminal differentiation of IgG B cells. Blood.

[58] Diehl S.A., Schmidlin H., Nagasawa M., van Haren S.D., Kwakkenbos M.J., Yasuda E., Beaumont T., Scheeren F.A., Spits H. (2008). STAT3-mediated up-regulation of BLIMP1 is coordinated with BCL6 down-regulation to control human plasma cell differentiation. J. Immunol..

[59] Avery D.T., Deenick E.K., Ma C.S., Suryani S., Simpson N., Chew G.Y., Chan T.D., Palendira U., Bustamante J., Boisson-Dupuis S. (2010). B cell-intrinsic signaling through IL-21 receptor and STAT3 is required for establishing long-lived antibody responses in humans. J. Exp. Med..

[60] Deenick E.K., Avery D.T., Chan A., Berglund L.J., Ives M.L., Moens L., Stoddard J.L., Bustamante J., Boisson-Dupuis S., Tsumura M. (2013). Naive and memory human B cells have distinct requirements for STAT3 activation to differentiate into antibody-secreting plasma cells. J. Exp. Med..

[61] Carbone G., Wilson A., Diehl S.A., Bunn J., Cooper S.M., Rincon M. (2013). Interleukin-6 receptor blockade selectively reduces IL-21 production by CD4 T cells and IgG4 autoantibodies in rheumatoid arthritis. Int. J. Biol. Sci..

[62] Cogliati S., Frezza C., Soriano M.E., Varanita T., Quintana-Cabrera R., Corrado M., Cipolat S., Costa V., Casarin A., Gomes L.C. (2013). Mitochondrial cristae shape determines respiratory chain supercomplexes assembly and respiratory efficiency. Cell.

[63] Hackenbrock C.R. (1966). Ultrastructural bases for metabolically linked mechanical activity in mitochondria. I. Reversible ultrastructural changes with change in metabolic steady state in isolated liver mitochondria. J. Cell Biol..

[64] Gomes L.C., Di Benedetto G., Scorrano L. (2011). During autophagy mitochondria elongate, are spared from degradation and sustain cell viability. Nat. Cell Biol..

[65] Wu M., Gu J., Guo R., Huang Y., Yang M. (2016). Structure of Mammalian Respiratory Supercomplex I1III2IV1. Cell.

[66] Gu J., Wu M., Guo R., Yan K., Lei J., Gao N., Yang M. (2016). The architecture of the mammalian respirasome. Nature.

[67] Letts J.A., Fiedorczuk K., Sazanov L.A. (2016). The architecture of respiratory supercomplexes. Nature.

[68] Acin-Perez R., Fernandez-Silva P., Peleato M.L., Perez-Martos A., Enriquez J.A. (2008). Respiratory active mitochondrial supercomplexes. Mol. Cell.

[69] Althoff T., Mills D.J., Popot J.L., Kuhlbrandt W. (2011). Arrangement of electron transport chain components in bovine mitochondrial supercomplex I1III2IV1. EMBO J..

[70] Vukotic M., Oeljeklaus S., Wiese S., Vogtle F.N., Meisinger C., Meyer H.E., Zieseniss A., Katschinski D.M., Jans D.C., Jakobs S. (2012). Rcf1 mediates cytochrome oxidase assembly and respirasome formation, revealing heterogeneity of the enzyme complex. Cell Metab..

[71] Moreno-Lastres D., Fontanesi F., Garcia-Consuegra I., Martin M.A., Arenas J., Barrientos A., Ugalde C. (2012). Mitochondrial complex I plays an essential role in human respirasome assembly. Cell Metab..

[72] Chen Y.C., Taylor E.B., Dephoure N., Heo J.M., Tonhato A., Papandreou I., Nath N., Denko N.C., Gygi S.P., Rutter J. (2012). Identification of a protein mediating respiratory supercomplex stability. Cell Metab..

[73] Shulman Z., Gitlin A.D., Weinstein J.S., Lainez B., Esplugues E., Flavell R.A., Craft J.E., Nussenzweig M.C. (2014). Dynamic signaling by t follicular helper cells during germinal center B cell selection. Science.

[74] Petersen O.H., Verkhratsky A. (2016). Calcium and ATP control multiple vital functions. Philos. Trans. R. Soc. Lond. B Biol. Sci..

[75] Geltink R.I.K., Kyle R.L., Pearce E.L. (2018). Unraveling the complex interplay between T cell metabolism and function. Annu. Rev. Immunol..

[76] Van der Windt G.J., Everts B., Chang C.H., Curtis J.D., Freitas T.C., Amiel E., Pearce E.J., Pearce E.L. (2012). Mitochondrial respiratory capacity is a critical regulator of CD8+ T cell memory development. Immunity.

[77] Allard E.L., Hardy M.P., Leignadier J., Marquis M., Rooney J., Lehoux D., Labrecque N. (2007). Overexpression of IL-21 promotes massive CD8+ memory T cell accumulation. Eur. J. Immunol..

[78] Zeng R., Spolski R., Finkelstein S.E., Oh S., Kovanen P.E., Hinrichs C.S., Pise-Masison C.A., Radonovich M.F., Brady J.N., Restifo N.P. (2005). Synergy of IL-21 and IL-15 in regulating CD8+ T cell expansion and function. J. Exp. Med..

[79] Furtek S.L., Backos D.S., Matheson C.J., Reigan P. (2016). Strategies and approaches of targeting STAT3 for cancer treatment. ACS Chem. Biol..

[80] Huynh J., Etemadi N., Hollande F., Ernst M., Buchert M. (2017). The JAK/STAT3 axis: A comprehensive drug target for solid malignancies. Semin. Cancer Biol..

[81] Hong D., Kurzrock R., Kim Y., Woessner R., Younes A., Nemunaitis J., Fowler N., Zhou T., Schmidt J., Jo M. (2015). AZD9150, a next-generation antisense oligonucleotide inhibitor of STAT3 with early evidence of clinical activity in lymphoma and lung cancer. Sci. Transl. Med..

[82] Leong P.L., Andrews G.A., Johnson D.E., Dyer K.F., Xi S., Mai J.C., Robbins P.D., Gadiparthi S., Burke N.A., Watkins S.F. (2003). Targeted inhibition of Stat3 with a decoy oligonucleotide abrogates head and neck cancer cell growth. Proc. Natl. Acad. Sci. USA.

[83] Yue P., Turkson J. (2009). Targeting STAT3 in cancer: How successful are we?. Expert Opin. Investig. Drugs.

[84] Zhang X., Yue P., Page B.D., Li T., Zhao W., Namanja A.T., Paladino D., Zhao J., Chen Y., Gunning P.T. (2012). Orally bioavailable small-molecule inhibitor of transcription factor Stat3 regresses human breast and lung cancer xenografts. Proc. Natl. Acad. Sci. USA.

[85] Turkson J., Kim J.S., Zhang S., Yuan J., Huang M., Glenn M., Haura E., Sebti S., Hamilton A.D., Jove R. (2004). Novel peptidomimetic inhibitors of signal transducer and activator of transcription 3 dimerization and biological activity. Mol. Cancer Ther..

[86] Bharadwaj U., Eckols T.K., Xu X., Kasembeli M.M., Chen Y., Adachi M., Song Y., Mo Q., Lai S.Y., Tweardy D.J. (2016). Small-molecule inhibition of STAT3 in radioresistant head and neck squamous cell carcinoma. Oncotarget.

[87] Ogura M., Uchida T., Terui Y., Hayakawa F., Kobayashi Y., Taniwaki M., Takamatsu Y., Naoe T., Tobinai K., Munakata W. (2015). Phase i study of OPB-51602, an oral inhibitor of signal transducer and activator of transcription 3, in patients with relapsed/refractory hematological malignancies. Cancer Sci..

[88] Wong A.L., Soo R.A., Tan D.S., Lee S.C., Lim J.S., Marban P.C., Kong L.R., Lee Y.J., Wang L.Z., Thuya W.L. (2015). Phase I and biomarker study of OPB-51602, a novel signal transducer and activator of transcription (STAT) 3 inhibitor, in patients with refractory solid malignancies. Ann. Oncol..

[89] Miyoshi K., Takaishi M., Nakajima K., Ikeda M., Kanda T., Tarutani M., Iiyama T., Asao N., DiGiovanni J., Sano S. (2011). Stat3 as a therapeutic target for the treatment of psoriasis: A clinical feasibility study with STA-21, a Stat3 inhibitor. J. Investig. Dermatol..

[90] Rubbert-Roth A., Furst D.E., Nebesky J.M., Jin A., Berber E. (2018). A review of recent advances using tocilizumab in the treatment of rheumatic diseases. Rheumatol. Ther..

[91] Schwartz D.M., Kanno Y., Villarino A., Ward M., Gadina M., O’Shea J.J. (2017). JAK inhibition as a therapeutic strategy for immune and inflammatory diseases. Nat. Rev. Drug Discov..

[92] Dhillon S. (2017). Tofacitinib: A review in rheumatoid arthritis. Drugs.

